# Mechanisms employed by retroviruses to exploit host factors for translational control of a complicated proteome

**DOI:** 10.1186/1742-4690-6-8

**Published:** 2009-01-24

**Authors:** Cheryl Bolinger, Kathleen Boris-Lawrie

**Affiliations:** 1 Center for Retrovirus Research, Department of Veterinary Biosciences, Molecular, Cellular, and Developmental Biology graduate program, The Ohio State University, Columbus, Ohio, USA

## Abstract

Retroviruses have evolved multiple strategies to direct the synthesis of a complex proteome from a single primary transcript. Their mechanisms are modulated by a breadth of virus-host interactions, which are of significant fundamental interest because they ultimately affect the efficiency of virus replication and disease pathogenesis. Motifs located within the untranslated region (UTR) of the retroviral RNA have established roles in transcriptional trans-activation, RNA packaging, and genome reverse transcription; and a growing literature has revealed a necessary role of the UTR in modulating the efficiency of viral protein synthesis. Examples include a 5' UTR post-transcriptional control element (PCE), present in at least eight retroviruses, that interacts with cellular RNA helicase A to facilitate cap-dependent polyribosome association; and 3' UTR constitutive transport element (CTE) of Mason-Pfizer monkey virus that interacts with Tap/NXF1 and SR protein 9G8 to facilitate RNA export and translational utilization. By contrast, nuclear protein hnRNP E1 negatively modulates HIV-1 Gag, Env, and Rev protein synthesis. Alternative initiation strategies by ribosomal frameshifting and leaky scanning enable polycistronic translation of the cap-dependent viral transcript. Other studies posit cap-independent translation initiation by internal ribosome entry at structural features of the 5' UTR of selected retroviruses. The retroviral armamentarium also commands mechanisms to counter cellular post-transcriptional innate defenses, including protein kinase R, 2',5'-oligoadenylate synthetase and the small RNA pathway. This review will discuss recent and historically-recognized insights into retrovirus translational control. The expanding knowledge of retroviral post-transcriptional control is vital to understanding the biology of the retroviral proteome. In a broad perspective, each new insight offers a prospective target for antiviral therapy and strategic improvement of gene transfer vectors.

## Introduction

**Figure 1:**
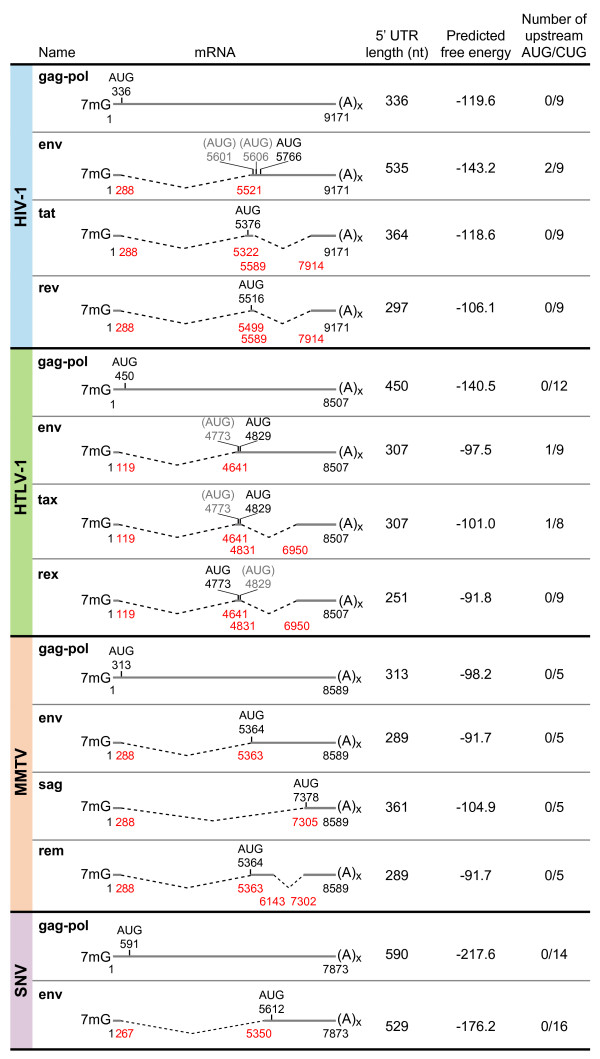
**Properties of selected retrovirus transcripts**. HIV-1, human T-cell leukemia virus type 1 (HTLV-1), mouse mammary tumor virus (MMTV), and spleen necrosis virus (SNV) transcripts are depicted, including predominant unspliced and spliced mRNA species. Numbering is in reference to the first nucleotide of R, the RNA start site, as +1. Red numbers below each mRNA indicate the nucleotide position of exon junctions. Dashed lines denote introns. AUG indicates translation initiation codon, and black numbers indicate AUG nucleotide positions. The unused AUG in bicistronic transcripts is depicted in gray parentheses. Predicted free energy values are derived from possible RNA structure calculated by Zuker mfold software version 3.2. The number of AUG or CUG codons upstream of the authentic AUG initiator codon is indicated in the far-right column. 7 mG, 5' RNA cap structure; (A)_x_, poly A tail. HIV-1 information was derived from [15]; HTLV-1 information was derived from [24] and GenBank NC_001436; MMTV information was derived from GenBank U40459, DQ223969, and [25]; SNV information was derived from reference sequence pPB101 [26].

Translation of mRNA is a multi-step process essential to all life. The ability of an organism to regulate mRNA translation represents a rapid, potent and strategic mechanism to control gene expression. Defects in translational regulation can be deleterious to survival. Three phases of translation include initiation, elongation and termination, with initiation considered the rate-limiting step. According to the ribosome scanning model of initiation, the mRNA template becomes activated for translation upon recognition of the 7-methyl-guanosine cap by eIF4E cap-binding protein, which complexes with other cytoplasmic initiation factors including eIF4G and eIF4A and eIF4B [1,2]. The 40S ribosomal subunit associates with eIF3 and the ternary complex (eIF2, GTP, Met-tRNA). This 43S charged ribosome complex joins the activated mRNA and scans in a 5'-3' direction until an initiator AUG codon in appropriate Kozak consensus context is detected ([1,3]). The 60S ribosomal subunit joins the complex to form the 80S complex and translation elongation ensues (for general translation review, see [2]). Transcripts containing a short (<100 nt), relatively unstructured 5' untranslated region (UTR) are generally good candidates for efficient ribosome scanning [4]. Conversely, transcripts that contain a longer and highly structured (free energy < -50 Kcal/mol) 5' UTR are less efficiently scanned [4]. The structural features of 5' UTR, and possibly features of the ribonucleoprotein complex (RNP), impede ribosome scanning and reduce the efficiency of translation initiation. Retrovirus proteins are synthesized from capped transcripts that uniformly contain long, highly structured 5' UTRs (Figure 1). Given this inhibitory characteristic, alternative mechanisms are expected to govern retrovirus translation. Investigation of mRNA translation in the retroviral model system has informed our understanding of virus-host interactions important for virus replication. These insights have also informed our understanding of specialized mechanisms that modulate translation of complex host cell mRNA templates.

### A dual fate for unspliced retroviral mRNA: translation, encapsidation, or both?

In the cytoplasm, the retroviral primary transcript (pre-mRNA) plays a dual role as unspliced mRNA template for translation and as genomic RNA that is encapsidated into assembling virions [5]. The RNA packaging signal in the 5' UTR of retroviral mRNA represents a pendulum that balances these possible fates of the genome-length RNA [5].

Results of *in vitro *translation assays determined that Gag can modulate translation of a reporter RNA that contains the HIV-1 5' UTR [6]. The translational output of the transcript was increased in response to low concentrations of Gag and reduced in response to high concentrations of Gag. Similar trends were observed in transient transfection assays. The results suggested bimodal modulation of translation by interaction between Gag and the HIV-1 5' UTR. The implicit mechanism is that Gag binds to the 5' RNA packaging signal and facilitates genome encapsidation at the expense of translation (Table 1) [6].

**Table 1:** Retrovirus mechanisms to modulate protein synthesis

**Mechanism**	**Examples of viruses reported to utilize mechanism^a^**	**Effect on translation**
**Internal Ribosome Entry Site** **(IRES)**	HIV-1, HIV-2, SIV, HMSV, MLV, RSV	Cap-independent translation enhancer. Ribosomes plus a subset of initiation factors internally initiate translation independently of a 5' 7-methylguanosine cap.

**Post-transcriptional control element (PCE)**	SNV, REV-A, HTLV-1, BLV, MPMV, FeLV, HIV-1, HFV	Novel 5' terminal cap-dependent translation enhancer. Specific interaction with RNA helicase A facilitates polysome loading and efficient viral protein production. PCE is not an IRES.

**Leaky scanning**	HIV-1	Readthrough of upstream AUG codons allows translation initiation of a downstream gene (i.e. *vpu *and *env*).

**Ribosome reinitiation**	RSV	Short upstream open reading frames present in 5' leader RNA attenuate translation initiation at the authentic gag-pol AUG. Effect is dependent on distance from AUG.

**Frameshifting**	Most retroviruses	Stimulatory signal and slippery sequence present in mRNA induce ribosome pausing and a -1 reading frame change. Results in translation of gag-pol open reading frame to produce reverse transcriptase and other enzymatic proteins.

**Termination codon readthrough**	FeLV, MLV	Termination codon of gag open reading frame is read as glutamate. Results in translation of gag-pol open reading frame to produce reverse transcriptase and other enzymatic proteins.

**Ribosome shunt**	Not determined	Scanning ribosome bypasses mRNA structural motif to reach AUG.

**Gag-gag mRNA interaction**	RSV, HIV-1	Gag protein binds to the 5' UTR of gag mRNA and attenuates translation efficiency.

***Cis*-acting repressive sequences/inhibitory sequences** **(CRS/INS)**	HIV-1	AU-rich sequences present in gag, pol and env mRNA bind cellular proteins involved in mRNA metabolism and translation. This association represses cytoplasmic expression of the mRNA.

**Rev**	HIV-1	Viral regulatory protein recognizes intronic *cis*-acting Rev response element (RRE) and counteracts repression by INS/CRS. Trans-activates nuclear export, with coincide increases in mRNA stability and polysome loading that result in robust viral protein production. HTLV-1 Rex/RxRE and MMTV Rem/RmRE activity activate nuclear export and may likewise enhance translational output.

^a ^BLV, bovine leukemia virus; FeLV, feline leukemia virus; HFV, human foamy virus; HMSV, Harvey murine sarcoma virus; HTLV-1, human T-cell leukemia virus type 1; MLV, murine leukemia virus; MPMV, Mason-Pfizer monkey virus; REV-A, reticuloendotheliosis virus strain A; RSV, Rous sarcoma virus; SIV, simian immunodeficiency virus; SNV, spleen necrosis virus.

A long-standing issue in retrovirus biology is whether or not the processes of gag mRNA translation and virion precursor RNA encapsidation are mutually exclusive [5]. The take-home message differs between retroviruses. For example, HIV-2 has been shown to encapsidate RNA co-translationally [7], while murine leukemia virus (MLV) produces two functionally distinct pools of mRNA to be used for either translation or virion assembly [8,9]. In the case of HIV-1, unspliced RNA can be used interchangeably for translation and virion assembly [9,10]. In distinction from HIV-2, translation is not a prerequisite to qualify unspliced HIV-1 RNA for packaging into virions [10].

LeBlanc and Beemon used translation-dependent nonsense mediated decay (NMD) as an innovative approach to evaluate this issue for Rous sarcoma virus (RSV) [11]. Their study evaluated RSV molecular clones that contain artificial pre-mature termination codons (PTC). The experiments determined that unspliced PTC-containing RSV RNA, which is a substrate for translation-dependent NMD, could be packaged into virions. A follow-up study in the context of the authentic provirus determined that RSV utilizes a 3' UTR RNA stability element to evade NMD and ensure appropriate levels of gag mRNA for virion protein synthesis [12]. The finding that unspliced RSV transcript can be a substrate for both translation and packaging into virions indicated that these processes are not mutually exclusive in this alpharetrovirus. A comprehensive review of the relationship between gag translation and virion precursor RNA packaging is presented elsewhere [5].

### Potential for alternative translation initiation

The 5' UTR of retroviral gag pre-mRNA contains a collection of highly conserved *cis*-acting sequences required for several steps in virus replication. For instance, the HIV-1 5' UTR contains the Tat trans-activation response element (TAR), primer binding site, genome dimerization signal, 5' splice site and a packaging signal [13]. Because some of these motifs are upstream of the 5' splice site, they are maintained within the 5' UTR of the ~30 alternatively spliced HIV-1 transcripts [14,15]. This proximal section of the 5' UTR has been shown to inhibit ribosome scanning and translation initiation of a reporter RNA [14-18]. In the context of the virus, ligation of the 5' exon to various distal exons produces additional species of complex 5' UTRs that are ~350 to 775 nucleotides in length (Figure 1). These long UTRs often contain AUG or CUG sequences upstream of the authentic initiator codon [19,20] (Figure 1), which interfere with translation initiation at the appropriate AUG [21]. Another complicating feature is that authentic initiator codons often are located within poor Kozak consensus sequences, which may provide another regulatory feature that modulates expression of the viral proteome (reviewed in [22]). For example, a weak Kozak sequence surrounding the HIV-1 vpu AUG promotes translation of the downstream *env *gene, a process referred to as leaky scanning [14,23]. The inhibitory features found in the HIV-1 5' UTR are also represented in all other retroviruses, as summarized for human T cell leukemia virus type 1 (HTLV-1), mouse mammary tumor virus (MMTV), and spleen necrosis virus (SNV) in Figure 1[15,24-26]. In spite of the multiple challenges to efficient cap-dependent translation initiation, sufficient retrovirus protein production prevails.

The potential dichotomy of mechanisms governing translation initiation of retroviruses is a topic of some controversy. The use of cap-independent initiation at an internal ribosome entry sequence (IRES) has been proposed to circumvent inhibition of scanning ribosomes by the complex 5' UTR. Originally identified in the *Picornaviridae*, which includes poliovirus and encephalomyocarditis virus (EMCV), the IRES promotes recruitment of the 43S ribosome independently of cap-binding [27-29]. Transcripts of the *Picornaviridae *lack a 5' cap and provide cytoplasmic viral enzymes to inactivate factors including eIF4E, eIF4G and poly(A) binding protein (PABP) [30-32]. Consequently, picornavirus transcripts are reliant on an IRES to initiate viral protein synthesis [27,28,33].

By contrast, transcripts of the *Retroviridae *are considered to bear a 5' cap and therefore IRES-dependent initiation is not necessarily critical. In support of this idea, translation of the avian spleen necrosis virus is reduced when cap-dependent translation is inhibited by infection with EMCV [34]. Nevertheless, the search for IRES activity in the *Retroviridae *has been extensive with IRES-like activity proposed for at least six retroviruses, including HIV-1 and HIV-2 [35-37], simian immunodeficiency (SIV) [38], RSV [39], and murine leukemia viruses (Friend and Moloney strains, F-MLV and MoMLV, respectively) [40-42], and Harvey murine sarcoma virus (HMSV) [43] (Table 1 and reviewed in [22]). Studies to identify internal initiation in isolated viral UTR segments have primarily utilized the transfection of bicistronic reporter plasmids. A caveat to this approach is false-positive IRES activity attributable to cryptic promoter activity or splicing of the test sequence. A case study of bicistronic reporter plasmids that employed extensive RNA analysis determined that 5' UTR sequences of HTLV-1, REV-A, or SNV produced multiple transcripts that correlated with false-positive IRES activity [34]. The false-positive activity was validated by the observation that transfection of homologous *in vitro *transcribed RNA did not recapitulate IRES activity. A possible caveat is that the transfected RNAs may fail to interact with necessary IRES-transacting factors (ITAFs) in the nucleus. An alternative approach to measure HIV-1 IRES employed poliovirus infection to inhibit cap-dependent translation initiation. The results determined that HIV-1 Gag protein synthesis is sustained from a heterologous reporter plasmid during poliovirus infection [36]. Unexpectedly, the putative IRES activity was conferred by sequences downstream of the gag translation initiation codon, rather than the 5' UTR. In summary, utilization of internal ribosome entry at retroviral IRES remains a controversial subject, and conditional IRES activity is an intriguing possible explanation for the disparate results. An alternative scenario is that features of the complex 5' UTR direct mechanistically uncharacterized virus-host interactions to modulate cap-dependent initiation. This scenario and its perspective into the translation of complex cellular mRNAs are discussed in the next section.

### Cap-dependent retrovirus translation enhancers

Retroviral RNA interacts with a collection of cellular and viral co-factors (see Table 2). Three examples of viral RNA-host protein interactions that facilitate retroviral translation will be discussed. These interactions offer the model that an active remodeling process balances appropriate viral RNA translation with efficient trafficking for RNA packaging into assembling virions.

**Table 2:** Retrovirus:host interactions involved in retroviral translation control

	**Host factor**	**Examples of interacting virus^a^**	**Effect on translation**
**Interacts with retrovirus protein & RNA**	Protein kinase R (PKR)	HIV-1, HTLV-1	HIV-1 Tat reduces PKR autophosphorylation. Tat and eIF2α compete as substrates of PKR. High levels of HIV-1 TAR RNA or HTLV-1 RxRE inhibit PKR autophosphorylation.
	
	Small RNA pathway components (Dicer & Drosha)	PFV, HIV-1	PFV Tas and HIV-1 Tat act as RNA silencing suppressors that combat the antiviral effect of small RNA pathway. Also miRNAs may be encoded by retroviruses that downregulate host antiviral defense.

**Interacts with retrovirus RNA**	TAR RNA binding protein (TRBP)	HIV-1	Binding of TRBP to HIV-1 TAR RNA results in increased HIV-1 transcription and translation.
	
	2', 5'-oligoadenylate-synthetase/RNaseL	HIV-1, HTLV-1	HIV-1 5' UTR RNA binds 2-5OAS resulting in RNAseL activity *in vitro*. HIV-1 infection is associated with reduced interferon production and reduced 2-5A:RNAseL binding, allowing HIV-1 mRNA to evade cleavage by RNaseL. HTLV-1 RxRE activates 2-5OAS *in vitro*.
	
	RNA helicase A (RHA or DHX9)	SNV, REV-A, HTLV-1, MPMV, HFV, FeLV, BLV, HIV-1	RHA binds PCE mRNA leading to increased polysome association and efficient protein synthesis.
	
	9G8	MPMV	In overexpression experiments, hyper-phosphorylated 9G8 binds constitutive transport element-containing reporter mRNA resulting in increased polysome accumulation and protein synthesis.
	
	Sam68, SLM-1, SLM-2	HIV-1, HTLV-1, EIAV, MPMV	Sam68, SLM-1 and SLM-2 act synergistically with HIV-1 Rev, HTLV-1 Rex and EIAV ERev to facilitate expression and proper cytoplasmic localization of RRE-containing mRNA. Sam68 also enhances translation of mRNA containing the MPMV constitutive transport element.
	
	hnRNP E1	HIV-1	hnRNPE1 binds HIV-1 mRNA at the exon splicing silencer in Rev exon (ESSE) and reduces Gag, Env, and Rev protein production.
	
	eRF1	MLV	MLV reverse transcriptase binds eRF1 promoting readthrough of the gag termination codon to produce proteins encoded by gag-pol.

^a ^BLV, bovine leukemia virus; EIAV, equine infectious anemia virus; FeLV, feline leukemia virus; HFV, human foamy virus; HTLV-1, human T-cell leukemia virus type 1; MLV, murine leukemia virus; MPMV, Mason-Pfizer monkey virus; REV-A, reticuloendotheliosis virus strain A; SNV, spleen necrosis virus.

#### Many retroviruses utilize a 5' terminal post-transcriptional control element responsive to cellular RNA helicase A

While cap-independent initiation at an IRES is one approach for viral mRNAs to overcome barriers to ribosome scanning, another is represented by the post-transcriptional control element (PCE) (Table 1). Similar to the IRES, the PCE initially was identified in viral mRNA and subsequently in cellular transcripts [34,44-47]. Accordingly, study of retroviral PCEs provides a window into translation control of complex cellular mRNAs [46].

PCE is a redundant stem-loop RNA structure that was initially identified in the 5' UTR of avian spleen necrosis virus (SNV) and subsequently in a growing collection of retroviruses across the metazoa including Mason-Pfizer monkey virus (MPMV), human foamy virus (HFV), reticuloendotheliosis virus strain A (REV-A), human T-cell leukemia virus type 1 (HTLV-1), feline leukemia virus (FeLV), bovine leukemia virus (BLV) [34,44,45,47-49] and HIV-1 (Bolinger and Boris-Lawrie, unpublished data). A significant step toward understanding the control mechanism enforced by PCE was the discovery that cellular RNA helicase A (RHA or DHX9) specifically associates with PCE and is critical for robust translation of PCE-containing mRNAs [46] (Table 2). RHA is a multifunctional DEIH box helicase and RNA binding protein, and deregulation of RHA has been associated with various cancers and autoimmune disease [50-53]. In addition, RHA knockout in mice is embryonic lethal [54]. Roles for RHA in transcription and pre-mRNA splicing have been characterized, and new evidence indicates a role for RHA in loading of guide-strand siRNA onto the RNA interference silencing complex (RISC) [55]. Hartman et al. determined that RHA interacts with PCE-containing mRNAs in the nucleus and cytoplasm, and postulated that RHA contributes to RNA/RNP remodeling that facilitates polyribosome association [46]. Upon RHA downregulation, PCE-containing mRNAs still accumulate in the cytoplasm, however they are translationally-silent and possibly sequestered in RNA storage granules. Likewise, non-functional PCE mutant RNAs accumulate in the cytoplasm; these transcripts lack efficient interaction with RHA and are poorly translated [44,46-48]. Experiments utilizing SNV PCE-HIV gag reporter RNA determined that RHA downregulation specifically decreased the rate of Rev/RRE-independent Gag protein production, independently of a change in global protein or RNA synthesis [46]. The effect of RHA on Gag production occurs at the post-transcriptional level because quantitative RNA analyses detected no significant change in steady-state gag mRNA levels or nuclear/cytoplasmic distribution.

The PCE/RHA RNA switch is also operative in human retroviruses. The R and U5 sequences of the HIV-1 and HTLV-1 5' LTR function coordinately to confer RHA-dependent PCE activity (Bolinger, Sharma, Singh, Boris-Lawrie, unpublished data). Experiments with HTLV-1 provirus indicated that downregulation of endogenous RHA significantly reduced polysome accumulation of HTLV-1 gag mRNA from 75% to ~10% [34]. Control experiments determined that RHA downregulation was specific to HTLV-1 gag and did not affect gapdh RNA or global cellular translation [34,46]. Experiments with HIV-1 provirus indicated that RHA downregulation reduces HIV-1 gag translation (Bolinger, Sharma, Singh, Boris-Lawrie, unpublished data). In summary, RHA/PCE operates a 5' proximal RNA switch that is critical for translation of many retroviruses (Tables 1 and 2).

#### Nuclear interaction with host proteins facilitates retrovirus translation

The post-transcriptional processes of mRNA splicing, export, and translation are mechanistically linked and unspliced host pre-mRNA is typically a poor substrate for nuclear export or cytoplasmic translation [56]. However, retroviruses utilize the unspliced pre-mRNA as template for synthesis of essential structural and enzymatic proteins. Retroviruses have therefore evolved specialized mechanisms to ensure efficient export and translation independently of cellular default controls. *Cis-*acting RNA elements and interactive partners, such as HIV-1 Rev responsive element (RRE) and Rev, HTLV-1 Rex responsive element (RxRE) and Rex, or Mason-Pfizer monkey (MPMV) virus constitutive transport element (CTE) are necessary for efficient nuclear export of unspliced viral pre-mRNA. While HIV-1 RRE or HTLV-1 RxRE interact with Rev or Rex to connect to the CRM1 export receptor, CTE of the genetically simpler MPMV directly interacts with the Tap/NXF1 nuclear export receptor [57-60]. Another nuclear protein, 9G8, is recruited to the MPMV CTE during transcription; subsequent dephosphorylation triggers recruitment of Tap/NXF1 [58,61-65]. The association of Tap/NXF1 with CTE is critical for the export of intron-containing MPMV mRNA into the cytoplasm, where it remains associated with 9G8 [59,60].

In a particular cellular environment CTE was shown to enhance the translational efficiency of HIV-1 gag-pol mRNA in conjunction with the PCE located in the MPMV 5' LTR [66]. The synergistic effect of PCE and CTE on protein production was observed in monkey COS cells, but not human 293 cells. Translation enhancement was also dependent on the presence of a retroviral promoter, which posited co-transcriptional recruitment of a cellular factor that is more available in Cos cells than 293 cells. Consistent with this idea, overexpression of Tap/NXF1 increased production of the Gag reporter protein by 30-fold in 293 cells independently of an increase in total gag mRNA abundance or cytoplasmic accumulation. The increase in translational utilization of the RNA was conferred specifically by CTE, since reporters containing PCE but not CTE, were not affected by Tap/NXF1 overexpression. These results provided an example of nuclear virus-host interaction that modulates translation.

In a separate study, splicing regulatory protein 9G8 was identified as a cellular protein that increases cytoplasmic utilization of the HIV-1 gag-pol-CTE reporter mRNA [66]. Overexpression of 9G8 in 293T cells produced a 10-fold increase in Gag protein production from the reporter RNA [60]. The overexpression of 9G8 did not alter cytoplasmic accumulation of the reporter RNA but increased polyribosome association by 10-fold. Ribosomal profiles and immunoblots indicated that hyperphosphorylated 9G8 was associated with high molecular weight complexes that were sensitive to EDTA treatment, indicating that 9G8 likely associated with polyribosomes and not other heavy complexes (referred to as "pseudo-polysomes" [67]). These results bolstered the recent realization that splicing regulatory proteins provide functional linkage between multiple steps of post-transcriptional gene regulation, and that the linkage is co-opted during retrovirus replication [68,69].

Sam68 (Src-associated in mitosis 68) is another host protein that increases cytoplasmic utilization of CTE-containing mRNA [70]. Sam68 has been shown to functionally synergize with Rev-like proteins of complex retroviruses to bolster viral post-transcriptional gene expression [71,72]. Sam68:RNA interaction in the nucleus has been shown to facilitate the association of viral RNA with translation machinery in the cytoplasm, resulting in enhanced protein production [73]. Sam68 activity is addressed in more detail below.

#### hnRNP E1 negatively influences HIV-1 protein production

RHA, 9G8, and Sam68 are examples of nucleocytoplasmic shuttling proteins that promote efficient viral protein production. By contrast, hnRNP E1 provides an antagonistic effect on HIV-1 protein synthesis. hnRNP E1 is a nuclear protein that interacts with the HIV-1 exon splicing silencer in Rev exon (ESSE3) [74]. Contrary to the name, this interaction is not associated with significant change in viral RNA splicing. Instead, overexpression of hnRNP E1 caused a substantial decrease in Gag (p55 and p24), Env (gp160/gp120), and Rev production. Fractionation assays indicated that the decreased level of Rev protein remained sufficient for export of HIV-1 Rev-dependent env RNA. A complementary experiment utilizing siRNA downregulation of endogenous hnRNP E1 recapitulated a significant change in Gag and Env protein without observable effect on RNA abundance or splicing. These results indicated that hnRNP E1 negatively affects HIV-1 translation. A possible mechanism is that hnRNP E1 blocks the association of the 60S ribosome with the 43S initiation complex once the initiation codon is reached, as has been observed for hnRNP E1 and *r15-LOX *mRNA [75]. Other feasible explanations are the disruption of another step in the translation process or the reduced stability of the nascent polypeptide.

### Initiation and beyond: Retrovirus protein production is reliant on ribosome frameshifting and leaky scanning

Retroviruses employ economical strategies of post-transcriptional control that culminate in expression of multiple viral open reading frames from a relatively small (~10 kb) genome. The strategies include alternative translation initiation, modulation of elongation and polypeptide termination.

#### Control of translation by RNA localization and leaky scanning

For genetically simple retroviruses, inefficient splicing produces Gag, Gag-Pol and Env open reading frames on separate transcripts. Genetically complex retroviruses, such as HIV-1, employ alternative splicing to produce open reading frames for regulatory and accessory proteins on additional transcripts (Figure 1) [14,15]. As summarized above, the 5' UTR of both unspliced and spliced retroviral transcripts contain features that impede ribosomes scan and alternative mechanisms are expected to choreograph viral protein production (reviewed in [22]). An economic strategy of leaky scanning provides HIV-1 Vpu and Env protein synthesis from a single bicistronic vpu-env mRNA [76]. Schwartz and colleagues demonstrated that translation of env is reliant on a weak Kozak consensus surrounding the vpu AUG. As a result, ribosomes scan past the vpu AUG to reach the env AUG and initiate translation of env, which is a process referred to as leaky scanning [23]. When the context of the vpu AUG is mutated to a match a strong Kozak consensus and reduce readthrough, env translation is abrogated [23]. By contrast, a higher level of env translation is achieved upon mutation of the vpu AUG [77]. Thus, leaky scanning through the vpu AUG is an important mechanism to achieve balanced expression of these HIV-1 gene products and is important for efficient virus replication [76].

A recent study of the 5' UTR of 16 env RNA isoforms produced by alternative splicing identified that production of Vpu is largely dependent on inclusion of exons that exclude the upstream rev AUG [78] These results reinforced the important role of the upsteam AUG in tempering env translation initiation [78]. Anderson and colleagues found that the four env mRNA isoforms containing exon 5E (isoforms 1,5,8, and 13) produced approximately four-fold more Vpu accessory protein in comparison to the rev AUG-containing isoforms. Mutation of the rev AUG in env2 increased Vpu production to a level similar to env1, indicating that initiation at the upstream rev AUG significantly depletes scanning ribosomes to initiate at the vpu initiation codon, consistent with the leaky scanning mechanism. However, in contrast to previous studies by Schwartz and colleagues, the presence of upstream AUGs had little effect on Env synthesis, suggesting the possibility of translation via a discontinuous scanning mechanism (IRES or ribosome shunt). The authors showed that the changes in protein expression were not attributable to aberrant transcripts from cryptic splicing, promoter activity or differential mRNA stability. A companion study by Krummheuer and colleagues suggested that a minimal five nucleotide open reading frame upstream of the vpu AUG acts as a ribosome pausing site, which is a feature of the ribosomal shunt characterized in cauliflower mosaic virus [79-84]. The cap-dependent ribosomal shunt is an intriguing alternative initiation mechanism that deserves further analysis in retroviruses.

#### Ribosomal frameshifting during translation elongation

Translation initiation has been studied intensively to understand mechanisms controlling cellular and viral protein synthesis. The translation elongation cycle, which is a closely regulated and high energy consuming process, also plays a profound role in the regulation of protein synthesis [85]. After initiation, phosphorylated elongation factors 1A and 1B (eEF1A and eEF1B) mediate amino acyl-tRNA recruitment to the ribosome A site and GDP/GTP exchange, respectively. Ribosome translocation occurs when elongation factor 2 (eEF2) associates with the ribosome and binds GTP to move the tRNA into the ribosome P site. This translocation results in a one-codon shift of the ribosome relative to the mRNA. mRNA containing a combination of repetitive sequences, referred to as slippery sequence, and stable secondary structure can pause ribosome locomotion leading to a mRNA reading frame change, referred to as programmed frameshift [86-88]. Essential retrovirus enzymatic proteins protease, integrase, and reverse transcriptase are encoded by the *pro *and *pol *genes that are distal to the Gag open reading frame in the viral unspliced mRNA. For lentiviruses and deltaviruses, the Pro and Pol coding regions are in a different reading frame than Gag. A -1 ribosome frameshift is necessary for synthesis of the Gag-Pro-Pol polyprotein, which is self-cleaved by the viral protease during maturation [87]. HIV-1 requires a single frameshift to produce Gag-Pol, while other retroviruses, such as HTLV-2, employ two separate frameshifts to produce Gag-Pro and Gag-Pro-Pol [89,90]. Frameshifting occurs at an approximate rate of 1 Gag-Pol for every 20 Gag molecules synthesized [25,87]. Disruption of either an upstream heptanucleotide slippery sequence (UUUUUUA in HIV-1) or an RNA stem-loop pseudoknot structure downstream of the frameshift site (referred to as the stimulatory signal) [91-93] alters frameshift efficiency and is deleterious to virus replication [94]. Recent work has indicated that both the seven nucleotides of the slippery sequence and the three preceding nucleotides are essential to maintain proper Gag-Pol ratio [95]. Brakier-Gingras and colleagues proposed an elegant model in which -1 frameshifting involves tRNA interaction with the ribosome at not only the P and A sites, but also with the E site [95]. Bicistronic reporters were constructed containing the HIV-1 seven nucleotide slippery sequence directly preceded by three nucleotides derived from the full 10 nucleotide slippery sequence of multiple viruses, including HTLV-1 and equine infectious anemia virus (EIAV). The upstream coding region contained renilla luciferase as a transfection efficiency control and firefly luciferase reporter downstream of the viral slippery sequence. Firefly Luciferase protein is observable in the event of a -1 ribosome frameshift. Results from transiently transfected 293T cells indicated that maintenance of the frameshift ratios required the authentic decanucleotide sequences from each virus. These results implicate that specific tRNA occupancy at the ribosome E site is important for -1 frameshift efficiency.

A similar reporter system was used to study the relationship between frameshifting and the activity of protein kinase R (PKR) [96]. This study found that inhibition of PKR activity by transfection of high levels of TAR RNA into Jurkat T cells or 293T cells resulted in decreased frameshifting efficiency. This effect occurred whether TAR was present in the reporter mRNA or expressed in *trans *from a separate plasmid. By contrast, activation of PKR by transfection of low amounts of TAR RNA increased frameshifting efficiency by 140%. TAR RNA had no effect on frameshifting after downregulation of PKR by siRNAs in 293T cells. Furthermore, the introduction of a TAR mutant deficient in PKR binding had no effect on frameshifting. The results indicated that TAR-PKR interaction contributes to efficient viral frameshifting. The proposed model is that translation initiation efficiency and frameshifting are inversely correlated. When the rate of translation initiation is slow (due to activation of PKR by TAR), frameshifting occurs at a higher rate because spacing between ribosomes increases and each ribosome encounters the frameshifting signal. Conversely, frameshifting decreases when the rate of initiation is increased because the stimulatory signal does not have time to refold and ribosomes continue to translate without pausing.

#### Modulation of translation termination by MLV reverse transcriptase

Translation termination occurs when the cellular release factor eRF1 recognizes a stop codon and GTP is hydrolyzed by eRF3 [97]. eRF1 has a structure similar to tRNA and is thought to bind the ribosome A site in a similar fashion to tRNA; it is proposed that stop codon recognition occurs through anticodon mimicry [98,99]. The hallmark enzyme of the retrovirus family, reverse transcriptase, is encoded by the *pol *gene and is absolutely essential for viral replication. For synthesis of infectious retrovirus, pol RNA is translated by a ribosomal frameshift at a slippery sequence (as in HIV-1) or by readthrough of the gag termination codon (as in FeLV and MLV) [87,100]. In the case of readthrough, a UAG stop codon is read as glutamine and translation proceeds to generate the Gag-Pol polyprotein [101,102]. A combination of yeast two-hybrid, *in vitro, *and *in vivo *studies by Orlova and colleagues demonstrated that MLV reverse transcriptase binds to eRF1 through a direct protein-protein interaction that enhances readthrough of the gag stop codon [103]. This interaction appears to be specific to MLV, as HIV-1 reverse transcriptase, which does not require termination codon readthrough for Pol synthesis, did not bind eRF1. The interaction of MLV RT with eRF1 allows the RT to self-regulate translation termination, thereby maintaining an appropriate ratio of Gag:Gag-Pol protein, which is critical to generate infectious virus [104].

### Retroviral regulatory export proteins Rev, Rex, and Rem may moonlight as translation stimulation factors

The HIV-1 post-transcriptional regulatory protein, Rev, is a 116 amino acid nuclear-cytoplasmic shuttling RNA binding protein [105-107] that is required for delivery of genome-length, unspliced RNA to the cytoplasm for subsequent translation and/or packaging into virions [5]. Synonymous loci have been identified in other complex retroviruses, including HTLV-1 and HTLV-2 Rex/RxRE and mouse mammary tumor virus (MMTV) Rem/RmRE [108-111].

Extensive experimentation with reporter plasmids and HIV-1 provirus has characterized Rev/RRE as a potent molecular switch that significantly trans-activates cytoplasmic accumulation of intron-containing RNAs. In the absence of Rev, cis-acting inhibitory sequences cause nuclear retention and low steady-state accumulation of these RNAs [112-116]. In the presence of Rev, the stability and nuclear export of RRE-containing transcripts are activated [117]. In addition to trans-activation of HIV-1 mRNA export, Rev has been identified to promote translation of RRE-containing mRNA [118,119]. D'Agostino et al. demonstrated that co-transfection of Rev with gag-RRE reporter plasmids in HeLa-Tat cells yielded a discordant relationship between the increase in cytoplasmic RNA and Gag protein levels. Addition of Rev caused a 4 to 16-fold increase in cytoplasmic accumulation of reporter RNA while Gag protein level increased by a discordant 850-fold. Ribosomal profile analysis indicated an increase in gag polysome association from 4% to 20% upon addition of Rev. The results indicated that Rev/RRE activity increases the cytoplasmic utilization of the Rev-dependent mRNA. A similar conclusion was reached by Arrigo et al., who transfected lymphoid cells with HIV-1 provirus that either lacked or contained the Rev open reading frame [118,120]. The presence of Rev increased polysome association of gag, vif, vpr, and vpu/env mRNA, indicating that Rev increases the translational efficiency of Rev-dependent transcripts. The observation by Cochrane and colleagues that Rev/RRE activity requires interaction with newly synthesized RNA [121] is consistent with the theme that nuclear interactions facilitate cytoplasmic utilization of retroviral RNA, as discussed above for RHA/PCE and 9GA/CTE.

A translational role for Rev-like proteins encoded by other retroviruses remains to be investigated in detail. Notably, study of HTLV-2 Rex-2/RxRE activity measured a 7-to-9 fold increase in cytoplasmic accumulation of gag RNA that was accompanied by a discordant 130-fold increase in steady state Gag protein, which is reminiscent of HIV-1 Rev/RRE activity [119,122]. Given the similarity of domain structure between HIV-1 Rev, HTLV-1 Rex and MMTV Rem, the conservation of functional activity in both nuclear export and cytoplasmic translation is an expectation (Table 2).

Study of the cellular protein Sam68 has produced additional insights into post-transcriptional control of retroviral gene expression. Sam68 and Sam68-like proteins SLM-1 and SLM-2 act synergistically with Rev to increase expression of RRE-containing mRNAs [73,123]. Synergistic activity is also observed by co-expression of Sam68 and HTLV-1 Rex or EIAV ERev [124]. Furthermore, C-terminal truncation of Sam68, which deletes the nuclear localization signal, generates an isoform that inhibits Rev activity and negated the effects of wild type Sam68, SLM-1 or SLM-2 on HIV-1 gene expression [73,123]. The cytoplasmically-restricted Sam68 mutant did not inhibit Rev shuttling ability, but changed the cytoplasmic distribution of RRE-containing unspliced HIV-1 env reporter RNA from a dispersed pattern to sequestration at the nuclear periphery. Addition of the SV40 large T antigen nuclear localization signal to the Sam68 mutant restored proper distribution of cytoplasmic RRE-RNA. These observations posited that interaction of Sam68 and RRE in the nucleus is critical for the target RNA to associate with translation machinery in the cytoplasm [73]. In sum, results with Rev, Sam68, RHA, 9G8 and hnRNPE1 echo the theme that nuclear interactions are important for productive retrovirus translation in the cytoplasm.

Rev/RRE regulation of viral protein production involves derepression of *cis*-acting repressive sequences (CRS, also referred to as instability sequences [INS]) present in HIV-1 gag, gag-pol, and env mRNA [112,115,125]. When placed 3' to chloroamphenicol acetyltransferase reporter gene, AU-rich segments of gag, pol, or env coding regions substantially reduced reporter gene activity [112,115,125]. Addition of the Rev responsive element (RRE) in *cis *and Rev protein in *trans *alleviated gene repression. In this reporter system, cytoplasmic accumulation and steady-state RNA levels were relatively unaffected, although the addition of CRS to a different reporter caused nuclear retention in the absence of Rev [112]. RNA affinity assays indicated that CRS interacted with cellular splicing factor hnRNPC1 and potentially other undefined factor(s). The nuclear retention of viral mRNA was attributed to sequestration in RNA-protein complexes that are inaccessible for nuclear export [126].

Further study of HIV-1 *cis*-acting repressive sequences by Schwartz et al (referred to as INS elements) showed that AU-rich regions of p17^gag ^transcript decreased mRNA stability and reduced the steady-state abundance of viral RNA [115]. Similar to experiments described above, co-transfection of Rev expression plasmid relieved the inhibitory effect of INS on HIV-1 tat-RRE-gag reporter RNA. Mutations in the INS lead to Rev-independent gene expression and eliminated the instability phenotype. Multiple proteins have been identified to bind INS elements, including hnRNP A1 [127,128], polypyrimidine tract binding protein (PTB) [127,128], polypyrimidine tract-binding protein-associated splicing factor (PSF) [129] and poly(A)-binding protein (PABP) [130]. The binding partners have been postulated to coordinate interrelated steps in HIV-1 RNA post-transcriptional control. For example, hnRNP A1 was found to stimulate Rev-dependent mRNA export [131]; PSF modulates nuclear retention [129]; and PABP modulates transcript stability [130]. Overexpression of PSF decreases the abundance of all HIV-1 transcripts, with the most substantial effect on unspliced and singly spliced species [129]. Binding of PABP to INS RNA correlated with reduced mRNA stability, which indicated that PABP association at regions other than the polyA tail could disrupt or compete the formation of RNP necessary for efficient translation initiation.

In addition to affecting HIV-1 RNA export and stability, codon bias that results from the unusually high AU content of HIV-1 transcripts may attenuate translation. The presence of rare codons has been demonstrated to induce ribosome pausing that culminates in RNA turnover (Reviewed by Hentze and Kulozik [132]). Codon optimization of HIV-1 env and gag-pol sequences has been shown to increase viral protein production in human cells [133,134]. Furthermore, codon optimization of gag-pol increased RNA abundance and eliminated the requirement for Rev/RRE in HIV-1 based vector systems [134]. The observations that codon optimization eliminates Rev/RRE dependence support a positive role for Rev/RRE in stability and translation of HIV-1 transcripts.

Although studies have observed CNS/INS to modulate HIV-1 RNA by different post-transcriptional mechanisms, the convergent conclusion is that these viral sequences interact with cellular proteins involved in splicing and other steps in mRNA metabolism to balance virus protein production. Furthermore, these studies emphasize that Rev/RRE is a multifunctional protein/RNA interaction which can trans-activate the stability of RRE-containing viral mRNAs, their export from the nucleus, and their translation in the cytoplasm. The combination of these roles makes Rev/RRE a strategic regulatory axis in HIV-1 replication.

### A balancing act: The ability of retroviruses to elicit or block innate host defense pathways

**Figure 2:**
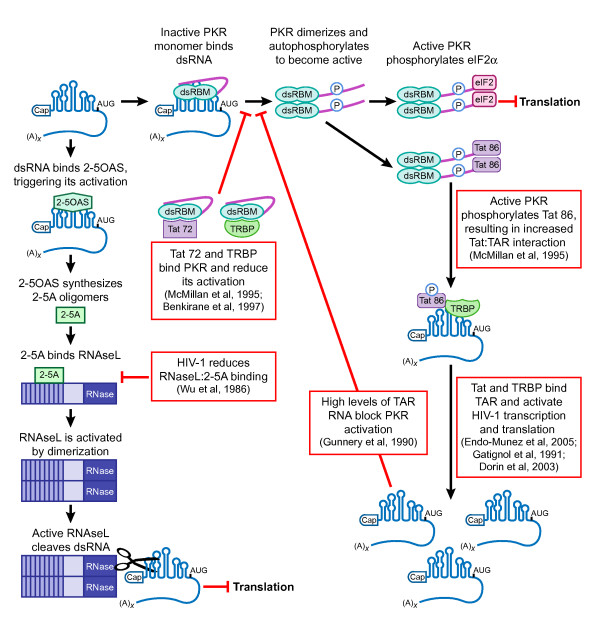
**HIV-1 modulates the interferon-induced antiviral host mechanisms, protein kinase R and 2',5'-oligoadenylate synthesis**. Structure in blue represents generic retrovirus transcript with highly structured 5' UTR. Left panel illustrates the 2',5'-oligoadenylate synthesis (2-5OAS) pathway that typically results in RNAseL activation and cleavage of viral double-stranded RNA. Vertical lines within RNaseL indicate ankyrin repeats. Right panel illustrates the double-stranded RNA-inducible PKR pathway. PKR is depicted by the green N-terminal double-stranded RNA binding motif (dsRBM) with central domain and C-terminal kinase domain depicted by pink line. Tat transactivation response element (TAR) RNA binding protein (TRBP), Tat, and 2-5OAS proteins are marked. Tat 86 and Tat 72 indicate the 86 amino acid and 72 amino acid isoforms of HIV-1 Tat. Circles labeled with P indicate phosphorylation. Black arrows indicate normal progression through the pathway. Red block arrows and text boxes outlined in red indicate points of interaction and modulation by HIV-1.

Studies have revealed retrovirus escape from multiple mechanisms of innate cellular defense and exploitation of these pathways for benefit of the virus. Strategies for exploitation are enacted by viral RNA elements, such as the HIV-1 Tat trans-activation response element (TAR), or through direct interaction of viral proteins (such as HIV-1 Tat) with innate defense proteins. Here we discuss retrovirus interaction with: i) the interferon-induced protein kinase R (PKR) pathway; ii) the interferon-induced 2'5'-oligoadenylate synthesis pathway; and iii) the small RNA pathway (Figure 2). Investigation has determined the interplay between virus and cellular defense mechanisms to be highly dependent on virus concentration and has uncovered elegant stratagies that temporally control these interactions for the overall benefit of virus survival.

#### HIV-1 vs. the PKR pathway

HIV-1 TAR is a highly-conserved stable RNA stem loop that interacts with Tat protein to regulate viral transcription [135]. TAR has been shown to negatively regulate translation by at least two mechanisms: i) inhibition of scanning ribosomes on HIV-1 mRNA [16-18], and ii) activation of the interferon-induced serine/threonine protein kinase R (PKR) when at a low concentration [136]. Active PKR is composed of two N-terminal double-stranded RNA binding motifs (dsRBM) and a C-terminal kinase domain. The intra-molecular interaction between the dsRBM and kinase domain form an inactive "closed" conformation (Figure 2) [137,138]. A change to an "open" conformation is triggered by association of PKR with double-stranded RNA (including HIV-1 TAR), leading to PKR dimerization and autophosphorylation [139-141]. In its active form, PKR phosphorylates Ser-51 on the α subunit of eukaryotic initiation factor 2 (eIF2α), which blocks eIF2α activation of the guanine exchange factor eIF2B (for a general review, see [142]). The disruption of eIF2α function by PKR ultimately reduces global cellular translation.

HIV-1 is among a variety of eukaryotic viruses that have evolved to circumvent this cellular defense mechanism. Roy and colleagues found that although low levels of TAR RNA bind and activate PKR [136], productive HIV-1 infection causes a decrease in PKR protein levels [143]. A separate study indicated that a high concentration of TAR-containing RNA can inhibit PKR activation, possibly by sequestration of the protein [144]. The double-stranded nature of the HTLV-1 Rex response element (RxRE) inflicted a similar fate for PKR, affecting PKR autophosphorylation that diminished with increasing concentration of RxRE-containing RNA [145].

The reduction in PKR activity observed by Roy and colleagues occurred with both HIV-1 infection and expression of HIV-1 Tat alone, implicating Tat as a major contributor to the downregulation [143]. The action of Tat was specific to PKR, since another interferon-response pathway, 2',5'-oligoadenylate synthesis (2-5OAS), was not affected by Tat alone. Later *in vitro *work identified the basic region of Tat as a substrate of PKR, and found that Tat can compete with eIF2α for phosphorylation [146]. Pre-incubation of PKR with increasing concentrations of Tat protein in *in vitro *kinase assays showed that Tat effectively reduced autophosphorylation of PKR in response to dsRNA. This study indicated that both the one exon (Tat 72) and two exon (Tat 86) variants of Tat were phosphorylation substrates of PKR (72 and 86 refer to predicted Tat proteins in their amino acid lengths). McMillan and colleagues found that only Tat 86 could be phosphorylated by pre-activated PKR although both Tat variants interacted with PKR [147]. While Tat 72 was not a phosphorylation substrate for PKR, *in vitro *pulldown and kinase assays indicated that Tat 72 binds to PKR and acutely inhibits its autophosphorylation and its ability to phosphorylate Tat 86. *In vivo *interaction between PKR and Tat 72 was verified in both a HeLa cell line and an in HIV-1 infected T cells. Overexposure of immunoblots indicated a low, yet detectable interaction between PKR and Tat 86, consistent with Tat 86 being a substrate of PKR. Although not demonstrated, it seems likely that the interactions between PKR and Tat variants occur in the context of TAR RNA due to their shared ability to bind TAR. Separate work has determined that phosphorylation of Tat 86 by PKR increases its interaction with TAR RNA and triggers more robust transcription of viral RNA [148]. An intriguing possibility is that the Tat variants are temporally controlled during the HIV-1 lifecycle to strategically modulate viral protein production. Overall, the analyses of HIV-1/PKR interplay indicate that although the HIV-1 TAR structure should elicit activation of the PKR antiviral defense (which it does at low concentration), Tat uses two strategies to combat this action: blocking the autophosphorylation of PKR; or acting as a substrate competitor to reduce the level of phosphorylated eIF2α thereby altering global translation. An overview of HIV-1/PKR interplay is depicted in Figure 2.

An additional strategy to counter innate defense is the HIV-1 interaction with a cellular double-stranded RNA binding protein, TAR RNA binding protein (TRBP) (Table 2) [149]. TRBP (also designated TRBP1) was discovered to bind HIV-1 TAR in a cDNA screen in HeLa cells [149]. An isoform designated TRBP2 has been identified that includes a 21 amino acid extension at the N-terminus [150]. Because both TRBP isoforms have been reported to activate the HIV-1 LTR in human and murine cells, they appear to be functionally equivalent [151].

TRBP proteins facilitate HIV-1 production through at least two mechanisms: i) potently inhibiting interferon-α induced PKR autophosphorylation through a direct protein:protein interaction [152]; and ii) binding to HIV-1 TAR and Rev response element (RRE) RNA resulting in enhanced virus expression [149]. A protective activity of TRBP on HIV-1 was demonstrated by Benkirane et al., who found that CEM T cells infected with HIV-1 expressing TRBP1 were resistant to the antiviral effect of interferon-α treatment [152]. Interferon-α repression of HIV-1 replication is due largely to PKR activation; therefore, a role for TRBP proteins in the PKR pathway was investigated. Stable expression of TRBP1 in HeLa cells reduced phosphorylation of PKR in response to interferon-α. Co-immunoprecipitation assays indicated an RNA-independent interaction between TRBP1 and PKR; however, treatment with interferon-α reduced this interaction in a dose-dependent manner. Immunoblotting revealed that INF-α inversely affected steady-state levels of PKR (increased) and TRBP1 (decreased) proteins, which could explain the apparent reduction in protein-protein association. Separate studies have reported that HIV-1 infection reduces the production of interferon-α, -β, and -γ in both T cells and monocytes, which could be a cause for the correlated reduction of PKR activation in response to HIV-1 [153-155]. These strategic observations illuminate the vast web of interactions that HIV-1 can balance to sustain productive virus replication (Figure 2).

#### TRBP proteins directly modulate retrovirus translation

Structural features of TAR RNA reduce the translation efficiency of heterologous reporter mRNAs, and the introduction of point mutations that reduce the free energy of TAR restores translation of a TAR-CAT reporter RNA *in vitro *[17,156]. Addition of TRBP1 purified from *E. coli *caused a significant increase in translation of wild-type TAR-CAT RNA. Interestingly, both CAT and TAR-CAT RNAs elicited activation of PKR, as evidenced by the phosphorylation of eIF2α. This result suggested that the reduced translation of TAR-CAT mRNA and the subsequent rescue by TRBP1 was at least in part independent of a PKR-specific effect. Consistent with this idea, low concentrations of TRBP2 facilitated translation of TAR-Luciferase mRNA in transiently transfected PKR-deficient mouse embryonic fibroblasts [156]. Importantly, this experiment demonstrated that both TRBP1 and TRBP2 isoforms can impact the translation of TAR-containing RNAs. Experiments with truncated domains of TRBP2 showed that the double-stranded RNA binding domains singly or in combination were responsible for the stimulatory effect on reporter protein production. A comparison of reporter protein and mRNA levels showed that the effect induced by the TRBP proteins was attributable to increased translation and not increased steady-state mRNA levels. Taken together, these results provide an example of how HIV-1 RNA has evolved to utilize cellular proteins to boost translation (Table 2). A growing list of roles have been identified for TRBP proteins, including a key role in the small RNA pathway in a ribonucleoprotein complex with siRNA, Dicer, and Ago2 [157,158]. This and additional implications of TRBP activities on HIV-1 replication are discussed below and reviewed elsewhere [159].

#### HIV-1 versus the 2'–5' oligoadenylate pathway

Studies of infected patients have indicated that HIV-1 affects 2'–5' oligoadenylate synthetase (2-5OAS) (Figure 2), which is a key enzyme in the regulation of an additional interferon-induced antiviral defense. 2-5OAS binds dsRNA longer than 60 bases through its amino-terminal residues. This triggers 2-5OAS to synthesize 2',5'-oligoadenylate (2-5A), a small molecule that binds to ankyrin repeats in the enzyme RNaseL and causes its dimerization and activation [160,161]. Once activated, RNaseL cleaves viral dsRNA with the mission of blocking viral protein production. Multiple viruses, including HIV-1, have devised a strategy to cripple this host defense to promote virus survival [162-164]. Association of HIV-1 RNA with 2-5OAS was investigated by SenGupta and Silverman, who incubated affinity resins bound to either HIV-1 5' UTR RNA or poly r(I):r(C) with extracts of interferon-treated HeLa cells prior to activation of 2-5A synthesis by magnesium and ATP. Both RNAs bound 2-5OAS; however, 2-5A synthesis was lower by a factor of five in response to the HIV-1 RNA substrate [165]. A similar study found that *in vitro *transcribed HTLV-1 RxRE RNA stimulated 2-5A synthesis from both interferon-treated HeLa cells and assays that used purified 2-5OAS [145]. The level of 2-5A production in response to RxRE RNA was similar to that induced by TAR RNA, although poly r(I):r(C) RNA induced 2-5A synthesis more potently than either RxRE or TAR. The authors suggested that the increased ability of poly r(I):r(C) to induce 2-5A production relates to the vast size difference of the molecule compared to RxRE or TAR RNA; this size difference increases the interaction between 2-5OAS and dsRNA.

Although structures in the retroviral genome apparently trigger activation of the 2-5OAS pathway, the virus may stop RNA degradation at a later stage in the signaling cascade. In support of this idea, a 65% reduction in the binding affinity of 2-5A to RNase L was observed in lymphocytes of HIV-1-infected patients [153-155,166]. Conversely, recent work has found that activation of 2-5A activity can combat HIV-1 replication [167]. Homan and colleagues utilized a stable 2-5A agonist called 2-5A^N6B^, which is taken up into the cytoplasm of cultured T cells and subsequently activates RNaseL [167]. They found that treatment of PBMCs with 2-5A^N6B ^inhibited the generation of infectious HIV-1, increased the expression of interferon-α and -γ, and increased the expression of chemokines. Later work showed that 2-5A^N6B ^caused a significant decrease in HIV-1 Gag protein production at 96 hours post-infection of CD4+ lymphocytes cultured from healthy donors. This decrease also was observed in the supernatant of CD4+ and CD14+ cells isolated from HIV-1 seropositive patients [168]. Taken together, these results indicate that HIV-1 targets both the PKR and 2-5OAS antiviral pathways to protect viral RNA from degradation, which ultimately protects translation and the production of virus particles.

#### Retrovirus interplay with the small RNA pathway

The small RNA pathway operates a versatile innate antiviral defense, and retroviruses are likely to exploit some aspect of this antiviral defense [169]. MicroRNAs (miRNAs) are short non-coding regulatory RNAs (~21 to 25 nucleotides) expressed by viral genomes and their eukaryotic host [170,171]. The interaction of endogenously expressed miRNAs with distinct targeted mRNA potently down-regulates gene expression by triggering specific degradation or translational inhibition of the transcript. miRNAs have the potential to abrogate the deleterious effects of human retrovirus infection or at least slow disease progression [172-174]. While details of interplay between plant viruses and host RNA silencing are robust, details of retrovirus interplay with host RNA silencing are just beginning to develop.

Early evidence of host miRNA activity in retrovirus infection emerged from the finding that miR-32 downregulated primate foamy virus type 1 in human cells [170]. This study also identified that the PFV-1 Tas protein can act as an RNA silencing suppressor (RSS). RSSs play a well-described role in the pathogenesis of plant viruses, but remain a controversial phenomenon for animal viruses [175]. Interestingly, Tas RSS activity is recapitulated across the plant and animal kingdoms [170].

A role for RNA silencing in HIV-1 replication kinetics has been documented in both peripheral blood mononuclear cells [173] and in resting primary CD4+ T cells [174]. Mechanistically, host RNA silencing inhibits translation of HIV-1 gag-pol mRNA and possibly all HIV-1 transcripts [176]. This activity occurs independently of a change in steady state gag-pol mRNA and reflects an anti-viral activity of the host cell miRNAs. An examination of the 3' UTR of HIV-1 transcripts from several strains has identified binding sites for a cluster of cellular miRNAs [174]. The identified miRNAs are enriched in resting CD4+ T cells compared to activated CD4+ T cells. Inhibition of these miRNAs correlated with increased viral protein production and virus particle production in resting CD4+ T cells without altering the amount of spliced or unspliced HIV-1 mRNA. These results unveiled an important role for cellular miRNAs in HIV-1 latency.

HIV-1 has been shown to utilize several strategies to counter host RNA silencing: suppression, protection of viral RNA in viral nucleocapsid, evasion, modulation and adaptation to RNA silencing [177]. Suppression of miRNA-directed activity against HIV-1 was first documented by Bennasser et al. [178], but remains controversial [179]. Tat RSS activity is modest and not global, and is demonstrated in several [176,178] but not all assays [179]. Tat RSS activity is dependent on the double-stranded RNA binding domain [178], which is a feature conserved in many plant virus RSS [175]. Like PFV-1 Tas, HIV-1 Tat displays cross-kingdom RSS activity [176].

Multiple lines of evidence indicate a physiological role for RSS activity in productive expression of the HIV-1 provirus. First, downregulation of Dicer and/or Drosha, which suppressed the small RNA pathway, enhanced HIV-1 replication kinetics in chronically infected human PBMC and Jurkat T cells [173]. Second, plant virus RSS [176] or Ebola V35 [180] can replace the Tat RSS activity. Third, the attenuation in gag mRNA translation by RNA silencing is exacerbated by the K51A substitution in the Tat double-stranded RNA-binding domain [176,178]. Finally, when Dicer was down-regulated, this change rescued robust gag translation and bolstered HIV-1 virion production in a continuous cell line [176].

Additional evidence for HIV-1 modulation of the host small RNA pathway has been presented by Jeang and colleagues [158]. This study found that TAR:TRBP interaction effectively sequestered TRBP from Dicer. The outcome was a reduction in the processing of reporter shRNA (luciferase) and three endogenous pre-miRNAs (miR-16, miR-93, and miR-221) that are downregulated by HIV-1 [181]. The implicit consequence of TRBP sequestration by TAR is less TRBP is available to interact with Dicer and assist in loading of guide-strand miRNA into the RISC complex [158,182]. Taken together with the identification of Tat RSS activity, the results indicate redundant strategies operated by TAR RNA and Tat RSS protein temper host RNA silencing of HIV-1 gene expression.

In sum, the small RNA pathway represents yet another host post-transcriptional mechanism that the retrovirus leverages to its benefit. An interesting notion is that tissue-specific miRNAs may influence retrovirus evolution [183].

### Perspectives

In conclusion, investigations continue to unveil retrovirus-host interplay in post-transcriptional control. A recurrent theme is employment of viral strategies that leverage antiviral defenses to achieve balanced viral gene expression. The fundamental knowledge of retrovirus translational control continues to expand. Each new finding has potential utility to devising new strategies for antiviral therapy and improving retroviral vector transduction. A common theme of retrovirus translational control is that an RNA structural motif interacts with viral or cellular protein to modulate balanced levels of structural and enzymatic proteins necessary for viral replication (summarized in Tables 1 and 2). These RNA-protein interactions have already been employed to improve gene transfer vectors [184-186]. Further understanding of these RNA-protein interactions may ultimately generate specialized vectors or helper virus cassettes that modulate interactions with translation modulatory proteins, such as RNA helicase A, 9G8, or hnRNP E1, or other yet-to-be identified interaction partners.

Another significant arena is the understanding of viral proteins that alter cellular post-transcriptional innate defenses (summarized in Figure 2 and Table 2). A protein of obvious importance is HIV-1 Tat, which in addition to its essential role in virus-specific transcription, has the ability to block the antiviral PKR response and act as an RNA silencing suppressor. Given its multi-faceted activity, Tat could be a sophisticated target for antiviral therapy. The other HIV-1 regulatory protein Rev has already been targeted in clinical trial [187], but this approach to influencing viral translational control remains to be realized. Decoding the molecular mechanisms of retrovirus translational control is valuable to fundamental knowledge and to the development of targeted antiviral strategies and gene transfer tools.

## Competing interests

The authors declare that they have no competing interests.

## Authors' contributions

CB and KBL equally contributed to this work.
